# Predicting emotional responses in interactive art using Random Forests: a model grounded in enactive aesthetics

**DOI:** 10.3389/fpsyg.2025.1609103

**Published:** 2025-08-04

**Authors:** Xiaowei Chen, Zainuddin Ibrahim, Azlan Abdul Aziz

**Affiliations:** ^1^College of Arts, Zhejiang Shuren University, Hangzhou, China; ^2^College of Creative Arts, Universiti Teknologi MARA, Shah Alam, Malaysia; ^3^Faculty of Computer Science, Informatics, and Mathematics, Universiti Teknologi MARA, Melaka, Malaysia

**Keywords:** interactive art, emotional response, Random Forest, affective prediction, user experience, computational aesthetics

## Abstract

**Introduction:**

Interactive installation art offers immersive and participatory environments that elicit complex and multidimensional emotional experiences—encompassing sensorimotor engagement, affective resonance, and cognitive reflection. However, these emotional responses’ inherently dynamic, subjective, and often pre-reflective nature poses significant challenges to their systematic prediction and computational modeling.

**Methods:**

To address these challenges, the present study introduces an interpretable machine learning framework grounded in the Random Forest (RF) algorithm, which provides a balanced trade-off between predictive performance and model transparency, thereby aligning with the needs of theory-driven emotion research. Based on 390 valid questionnaire responses, emotional responses were operationalized along five distinct dimensions: bodily changes, sensory engagement, emotional connection, cognitive reflection, and active personalization. Predictor variables encompassed sensory stimuli, multimodal interactional features, and immersive environmental cues. Model evaluation was conducted using cross-validation and held-out test sets, applying classification and regression metrics to assess performance.

**Results:**

The RF model demonstrated the highest predictive accuracy in the domains of cognitive reflection (F1 = 0.746, accuracy = 0.769) and active personalization (F1 = 0.673, accuracy = 0.705), suggesting that these cognitively mediated responses exhibit greater consistency and learnability across participants. In contrast, bodily responses proved substantially less predictable (F1 = 0.379, accuracy = 0.397), likely due to their idiosyncratic, embodied, and non-verbal nature, which may not be adequately captured by self-report measures alone.

**Discussion:**

These differential results underscore the relative tractability of modeling reflective and agentic emotional states in contrast to those rooted in sensorimotor or affective processes. Moreover, the model’s consistent performance across all evaluation phases affirms its suitability as an exploratory tool for investigating emotion in interactive art contexts. This study contributes to the evolving convergence of affective computing, human-computer interaction (HCI), and empirical aesthetics. The proposed framework yields actionable insights for the design of emotionally adaptive systems. Future research should consider the integration of multimodal and temporally granular data, and the ethical dimensions associated with affective adaptivity in artistic and public-facing environments.

## 1 Introduction

Interactive installation art has increasingly emerged as a compelling medium for emotional engagement, offering immersive environments that dynamically respond to users’ presence, behavior, and decision-making. Enabled by rapid advancements in artificial intelligence (AI), sensor technologies, and real-time processing, although early installation works—such as Kaprow’s *The Yard* (1961)—already emphasized physical interaction and audience participation, contemporary interactive installations have evolved significantly, with a growing emphasis on the integration of real-time sensing technologies and AI-driven feedback mechanisms in order to enhance co-creative processes and deepen affective engagement (Obeidat, 2013; Patel et al., 2020; Cao et al., 2021; Raptis et al., 2021). Through the integration of computer vision and motion tracking, they generate responsive audiovisual feedback, inviting users into participatory experiences that are emotionally rich and sensorial layered (Raptis et al., 2021). However, despite growing scholarly and artistic interest, the mechanisms through which these environments evoke emotional responses remain underexplored. Much of the existing literature relies on qualitative interviews or retrospective self-reporting (Pelowski et al., 2018), offering limited insight into how specific interaction features influence emotional experience—or how these responses might be predicted in advance.

From a psychological standpoint, emotion in interactive art contexts is best understood as a dynamic and contextually contingent process rather than a discrete or static event. This study adopts Russell’s circumplex model of affect (Russell, 1980), which conceptualizes emotion along two continuous dimensions: valence (pleasantness) and arousal (activation level). This dimensional framework is particularly suited to capturing affective states’ subtle and evolving nature during immersive engagement. Compared with discrete emotion models—such as Ekman’s basic emotions framework—which categorize emotional states into fixed labels (e.g., joy, anger, fear), the circumplex model offers a more flexible structure that better accommodates the fluidity and transitional quality of emotions typically elicited in immersive, interactive settings.

Importantly, emotional experience in interactive installations is not merely received but enacted. Theories of enactive and participatory aesthetics (Varela et al., 1991; Gallagher, 2005; Savaş et al., 2021) posit that emotion is co-constructed through embodied interaction, interpretative exploration, and decision-based engagement. Users do not passively consume emotion—they actively generate and shape it. This perspective aligns with affective loop theory (Höök, 2008), which conceptualizes emotional expression as a recursive cycle between user actions and system feedback. In parallel, affective computing (Picard, 1997) has demonstrated the feasibility of real-time emotion detection and modeling by integrating physiological signals (e.g., EEG, EDA, PPG) and behavioral data. For instance, Becker et al. (2004) successfully applied multimodal sensing techniques in conversational agents—methods that offer valuable methodological parallels for emotionally modeling interactive art experiences. However, predictive modeling remains in its infancy within the interactive installation art domain. While descriptive and correlational studies abound, few attempts have been made to develop generalizable, theory-informed prediction frameworks. In response to this gap, recent literature has advocated for applying machine learning algorithms capable of capturing complex, nonlinear relationships in affective data (Chen and Ibrahim, 2023). Random Forests (RF) stand out for their robustness to noise, ability to model high-dimensional interactions, and interpretability through feature importance rankings (Meinshausen and Ridgeway, 2006; Breiman, 2001).

Accordingly, this study proposes a machine-learning framework based on Random Forests to model and predict emotional responses to interactive installation art. Drawing on 390 valid participant responses, we examine how interaction features—such as sensory stimulation, immersive context, and user agency—contribute to five key dimensions of emotional engagement: bodily changes, sensory involvement, emotional connection, cognitive reflection, and active personalization. To guide this investigation, we address the following research questions:

1. Which interaction features most significantly shape emotional responses in interactive installation art?
2. To what extent can Random Forest models accurately predict different dimensions of emotional response?
3. What theoretical and design insights can be derived from the resulting predictive patterns?

By integrating psychological theory with machine learning techniques, this research contributes to a more systematic understanding of emotion formation in interactive contexts. Furthermore, it offers practical implications for developing emotionally adaptive environments in fields such as exhibition design, art therapy, affective computing, and educational media.

## 2 Literature review

As critically examined by scholars such as Bishop (2005) and Reiss (2001), installation art has long moved beyond the static display paradigm, emphasizing spatial immersion and the co-agency of the viewer. From Kabakov’s total environments to contemporary interactive systems, the genre is characterized by its resistance to fixed formal boundaries, foregrounding embodiment, presence, and experiential engagement as central aesthetic strategies (Kabakov, 1995). Such an orientation toward lived experience has prompted further theoretical elaboration. For instance, Caldarola (2020) conceptualizes the immediacy of installation art as a phenomenological encounter, while Rebentisch and Hendrickson (2012, pp. 13–25, 39–57) offers a systematic philosophical analysis of its temporal and spatial dynamics. These accounts contribute to a broader discourse that transcends purely formalist or historically anchored interpretations. Interactive installation art has increasingly gained scholarly attention due to its capacity to evoke complex and multilayered emotional experiences through multisensory and participatory engagement. By combining visual, auditory, tactile, and conceptual stimuli, these artworks create conditions for embodied interaction and co-construction of meaning, positioning the viewer as an active participant rather than a passive observer (Savaş et al., 2021). Iconic works such as *Pulse Room* by Rafael Lozano-Hemmer and *Rain Room* by Random International exemplify this potential, utilizing real-time environmental feedback, spatial responsiveness, and bodily presence to induce affective states such as awe, curiosity, and introspection (Lozano-Hemmer, 2006; Random International, 2013).

This emotional depth has prompted increasing efforts to assess quantitatively, and model user affect in interactive art. While earlier studies primarily relied on qualitative self-report methods and post-hoc interviews (Pelowski et al., 2018), recent advances in affective computing have introduced machine learning as a powerful tool to predict emotional responses. Among these approaches, Random Forest (RF) algorithms have emerged as particularly well-suited for modeling affective phenomena, owing to their ability to handle nonlinear, high-dimensional, and noisy datasets with robustness and efficiency (Breiman, 2001). Given this expressive potential, researchers have increasingly sought to model the emotional impact of interactive art more systematically. In response to these modeling challenges and the need for interpretable prediction, supervised machine learning approaches have gained increasing traction in affective computing. Among them, Random Forests (RF) have attracted particular attention for their algorithmic transparency, adaptability, and robustness across heterogeneous datasets. RF models have demonstrated strong performance across diverse affective computing tasks, including emotion recognition from facial expressions (Gharsalli, 2015, 2016), speech prosody (Chen, 2020; Annal, 2022), and physiological signals such as EEG (Vasquez et al., 2023). Moreover, adaptive and personalized RF configurations—capable of dynamically adjusting to individual differences—have shown promise in enhancing cross-user generalizability, a critical feature for the heterogeneity of emotional reactions in art experiences (Gonzalez and Prevost, 2021).

Beyond the domain of affective computing, Random Forests have been widely adopted in fields such as landscape visualization (Zhou et al., 2018), clinical diagnostics (Maurya et al., 2023), and rural development analytics (Liang, 2022). These applications highlight the algorithm’s strength in managing moderate-scale datasets and providing interpretable decision structures. For emotionally adaptive art systems, such interpretability is essential—not only to support design interventions but also to trace the specific interactional variables (e.g., spatial customization, ambient lighting, narrative depth) that most significantly impact emotional outcomes. Although alternative models such as Support Vector Machines (SVMs) and deep learning architectures (e.g., CNNs, RNNs) have demonstrated impressive performance in emotion recognition tasks (Domingues et al., 2014; Hossain and Muhammad, 2019), their limitations—particularly in interpretability, data demand, and computational complexity—reduce their suitability for real-time or user-centered design applications. By contrast, RF models offer a balanced trade-off between accuracy, efficiency, and transparency, making them particularly suitable for interactive art contexts where datasets may be limited and design accountability is essential.

Nevertheless, the application of RF in modeling self-reported emotional states in interactive art remains limited. Existing studies predominantly emphasize biometric or audiovisual inputs, with insufficient attention to how discrete design features—such as multisensory feedback, spatial agency, or symbolic content—contribute to emotional variability. Bridging this gap is essential for advancing predictive modeling and theoretical understanding of emotional engagement in art. Addressing this design-feature gap is crucial for bridging computational modeling with practice-oriented art theory.

The present study applies Random Forest modeling to a dataset of 390 self-reported emotional responses collected from viewers of interactive installation artworks. This work contributes to the literature by demonstrating the effectiveness of Random Forests in modeling multiple dimensions of emotional engagement within immersive art contexts. Specifically, the model identifies key interaction features—sensory stimulation, immersive environment, and user control—that significantly shape users’ affective experiences. Furthermore, by integrating machine learning with psychological theory and aesthetic design principles, the study provides a framework for developing emotionally adaptive and user-sensitive systems. This is particularly valuable in applied contexts like real-time art installations or therapeutic environments, where interpretable and design-relevant insights must accompany predictive precision.

## 3 Methodology

### 3.1 Theoretical framework

This study is anchored in an integrative theoretical framework that draws from affective science, interactive aesthetics, and computational modeling to investigate and predict emotional responses elicited by interactive installation art. Specifically, three interrelated perspectives form the conceptual foundation: (1) dimensional emotion theory, (2) enactive and participatory aesthetics, and (3) affective computing within the domain of human-computer interaction. This comprehensive integration ensures theoretical coherence across levels of emotion representation, interaction mechanics, and computational modeling.

To conceptualize emotion, we adopt Russell’s circumplex model of affect (1980), which characterizes emotional states along two continuous dimensions—valence (pleasant–unpleasant) and arousal (activation–deactivation). Compared with discrete emotion models, this dimensional approach provides a flexible structure for capturing the nuanced and fluctuating emotional states commonly observed in immersive and interactive environments. Second, the framework is informed by enactive aesthetics (Varela et al., 1991; Gallagher, 2005) and participatory aesthetics (Savaş et al., 2021), which posit that affective experiences in art are not passively received but actively co-constructed through sensorimotor engagement, symbolic interpretation, and bodily participation. Users are thus conceptualized as agentive co-creators whose decisions, movements, and meaning-making strategies contribute directly to the emotional content of the artwork. To further articulate the complexity of embodied cognition within aesthetic contexts, this study draws on recent interdisciplinary scholarship that conceptualizes aesthetic experience as a dynamic, situated, and sensorimotor process. Enactivist and ecological frameworks, in particular, underscore the constitutive role of bodily engagement and environmental coupling in shaping affective responses (Tewes, 2022). Building on this foundation, Krueger (2021) elucidates mechanisms of empathy and entrainment, through which viewers become emotionally and physically attuned to artworks via sensorimotor synchronization. Complementing these theoretical accounts, Pelowski et al. (2023) offer empirical validation for the integration of cognitive and embodied states in aesthetic encounters, supported by findings from neuroimaging and psychological research, which reveal converging activation patterns in brain regions associated with sensorimotor integration and aesthetic appraisal. These theoretical and empirical contributions reinforce the embodied foundations of our study and recognize the relevance of complementary models such as reflective appraisal and predictive coding. Third, this study draws on principles from affective computing (Picard, 1997), which treats emotion as a quantifiable phenomenon that can be detected and modeled through behavioral, contextual, or physiological indicators. In particular, the affective loop model (Höök, 2008) underlines emotion as a dynamic feedback cycle between user input and system responsiveness—a process especially relevant in interactive art contexts. This theoretical refinement directly addresses concerns about embodied cognition’s conceptual and empirical robustness in our original framework.

**Figure 1:**
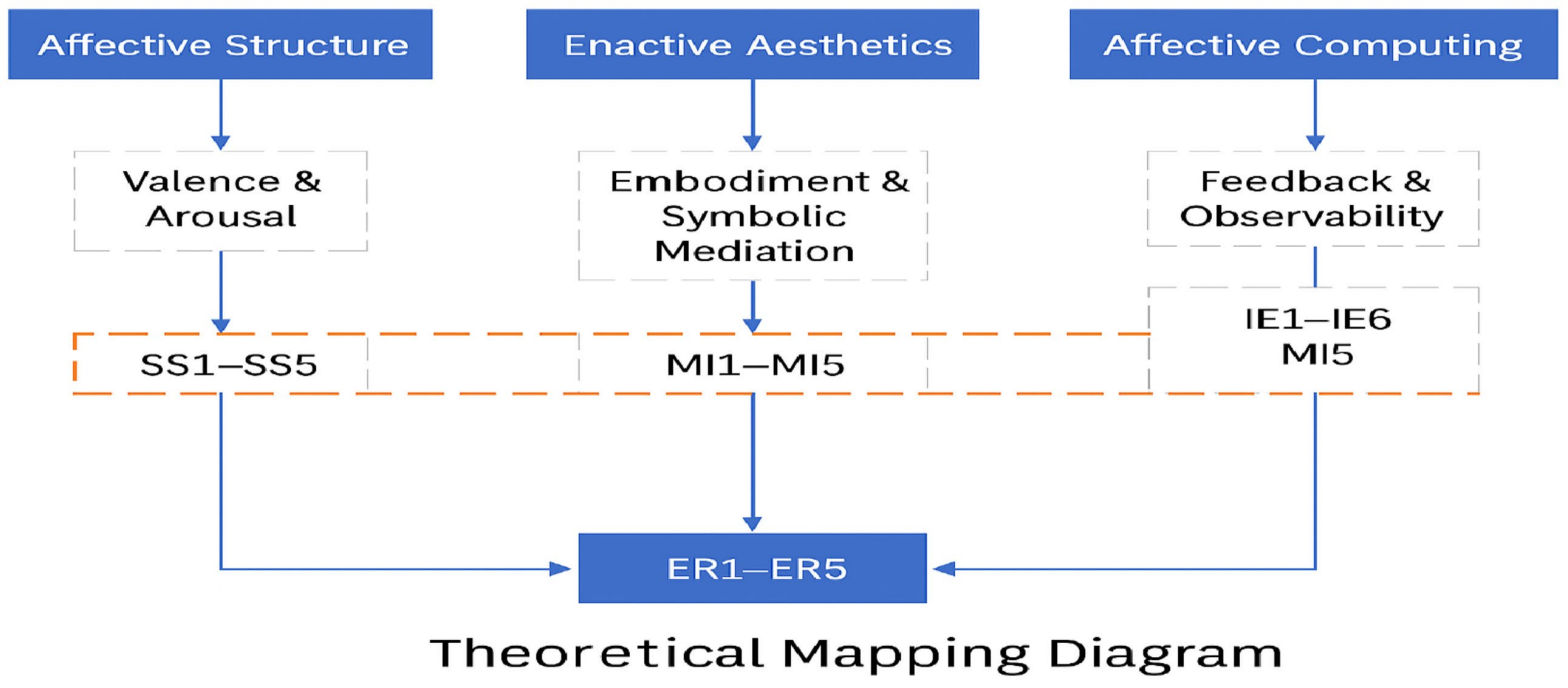
Theoretical mapping diagram. Variables are categorized according to their alignment with affective structure, enactive aesthetics, and affective computing. SS: sensory stimulus; MI: multidimensional immersion; IE: interactive engagement; ER: emotional response.

These theoretical foundations collectively inform the selection of independent variables—such as sensory stimulation, immersive environment, and interactive personalization—and justify using Random Forests (RF) as a modeling tool capable of capturing complex, nonlinear associations between interaction features and emotional outcomes. A theoretical mapping of constructs to variables is illustrated in Figure 1. This diagram visually connects each category of interaction features (SS, MI, IE) to the theoretical domains from which they are derived, offering a conceptual rationale for their role in predicting emotional responses (ER1–ER5). Figure 1 offers conceptual transparency and empirical grounding for the model’s structure by visually aligning variables with theoretical constructs.

These theoretical perspectives converge to form a multi-level model of affective engagement. At the foundational level, Russell’s circumplex model offers a structural framework for representing emotional outcomes (ER1–ER5) along valence and arousal. Building on this foundation, enactive and participatory aesthetics elucidate the mechanisms through which interaction unfolds, emphasizing bodily engagement, symbolic interpretation, and active personalization in the co-construction of affective meaning within interactive environments. In turn, affective computing—particularly the affective loop model—complements these perspectives by integrating emotional dynamics by modeling continuous feedback between user input and system response. Accordingly, the integrated framework aligns each theoretical perspective with a distinct functional layer: affective structure (SS), enactive aesthetics (MI), and interactional evaluation (IE) informed by affective computing. This layered architecture not only guides the selection of relevant predictor variables but also substantiates the application of Random Forest algorithms, which are well-suited for capturing nonlinear, high-dimensional relationships while providing interpretable insights into the relative contribution of each interaction feature to users’ emotional responses.

### 3.2 Survey design and data collection

Guided by the above conceptual foundations, we operationalized these constructs through a structured survey instrument to capture interaction features and emotional outcomes. To operationalize the theoretical model, we designed a structured online questionnaire to measure users’ emotional experiences after interacting with installation artworks. The instrument comprised three sections:

- Demographic Information: including age, gender, educational background, and previous exposure to art-related experiences.
- Interaction Features (Independent Variables): assessed using 31 items rated on a 5-point Likert scale, distributed across three dimensions—Sensory Stimulation (SS), Multidimensional Immersion (MI), and Immersive Environment (IE).
- Emotional Responses (Dependent Variables): assessed with five items, each corresponding to a specific affective outcome:
  - Emotional Response 1: Bodily Changes (e.g., increased heart rate, altered posture)
  - Emotional Response 2: Sensory Engagement
  - Emotional Response 3: Emotional Connection
  - Emotional Response 4: Cognitive Responses (e.g., reflection, interpretation)
  - Emotional Response 5: Active Participation and Personalization

This study employed a retrospective, experience-based survey approach targeting students from the School of Art and Design who had consistent exposure to interactive installation art through coursework, gallery visits, and institutional exhibitions. Rather than focusing on a single artwork, the questionnaire was designed to capture participants’ generalized emotional responses across various interactive installation experiences. This methodological choice was intended to enhance ecological validity by reflecting the diversity and complexity of affective impressions formed through real-world encounters with AI-driven installations. Data collection was conducted via Microsoft Forms. Of the 623 submitted questionnaires, 233 were excluded according to predefined criteria, including duplication and implausibly short completion times (i.e., less than 3 min). The final dataset consisted of 390 valid responses, yielding an effective response rate of 63%. Questionnaire items were either adapted from previously validated scales or developed by established conceptual frameworks to ensure theoretical alignment and construct validity. As shown in Table 1, each latent construct—Sensory Stimuli (SS), Multidirectional Interaction (MI), Immersive Environment (IE), and Emotional Response (ER)—was operationalized using specific measurement items, along with their corresponding sources.

**Table 1:** Latent variables and their corresponding measurement items.

Scale	Items	Source of scale
Sensory stimuli	Artificial intelligence-driven visual elements like adaptive lighting and dynamic color patterns appeal to me.	Jain and Ramakrishnan (2020)
	AI-driven auditory features, such as responsive soundscapes or music, create a deeply resonant and personalized experience for me.	
	Interacting with responsive haptic components in the unit, such as the touchscreen, dramatically increases my enjoyment.	
	The olfactory element with artificial intelligence responds to my actions by releasing smells, making my experience special and unforgettable.	
	Changing the tastes depending on my interactions will help me to have a rich sensory experience in the customized AI installation.	
	Including touch, sound, and sight in the exhibit helps me to connect with the work.	Developed by the author
	My emotional reaction is heightened by the installation’s numerous sensory experiences, which include sight and sound.	
	The whole approach of the artwork enriched my experience more than any regular art show.	
	Because of the numerous sensory inputs supplied by the AI-driven installation art, I developed a greater emotional connection to it.	
	The various sensory stimuli deepened my overall emotional connection to the art installation.	
Multidirectional interactive	Integrating visual, auditory, and tactile elements into the artwork immerses me more in the art.	Raptis et al. (2021)
	Multisensory interactions in the installation significantly increased my presence and immersion.	Velasco and Obrist (2021)
	My physical movements and behaviors, which influence AI functions like lighting, create a personalized experience.	Edmonds (2011)
	The layout and spatial design of the installation affect my level of interaction and emotional engagement.	Liu (2021)
	The diversified interface design within the artwork provides a richer, more compelling interactive experience.	De Bérigny et al. (2014)
	Story narratives embedded within the installation art enhance my emotional connection to the piece.	Anadol (2022)
	The use of metaphors and symbolism in the artwork deepens my understanding and emotional response.	
	The AI techniques used in the installation, from interactive colors to dynamic texture effects, greatly influenced my emotional response.	Developed by the author
	The real-time responsiveness and diversity of the artefacts allowed me to interact with the installation frequently, affecting my emotional response.	
	With AI, I can share digital experiences or co-engage with others, making my emotional experience even more prosperous.	
Immersive environment	My active participation in the installation, through movement or touch, deepens my sense of being within the art environment.	Schreuder et al. (2016)
	The degree to which I feel emotionally connected to the installation correlates with my sense of immersion.	Xu and Wang (2021)
	The more I engage with interactive features (like motion sensors or displays), the more immersed I feel in the artwork’s environment.	Developed by the author
	Personalization features of the installation, such as AI responses tailored to my actions, enhance my sense of immersion in the artwork.	Raptis et al. (2021)
	My immersion in the art installation is closely related to my emotional involvement and intensity of feelings.	Raptis et al. (2021)
	Features that enhance my sense of presence within the installation (such as interactive displays and motion sensors) directly influence my emotional responses.	Pavic et al. (2023)
	The technology increases my involvement by delivering rapid visual or aural feedback.	Developed by the author
	I could play longer and have more pleasure because it had game-like aspects, such as assignments or challenges.	
	How emotionally immersed I was in the event determined how long I interacted with the installation.	
	I felt ‘lost’ in the installation, as if detached from reality, which was a profound emotional experience.	
	The installation provided various ways of interaction that enriched my emotional journey through art.	
Emotional responds	There is a significant difference in the emotional experiences between one-dimensional and multidimensional interactions in AI-integrated art.	Konečni (2015)
	AI-integrated installation art that adapts and responds to my behavior helps produce a more profound emotional experience.	Akten et al. (2019)
	The responsive elements of the artwork broaden the variety of emotions I experience during interaction.	Brooks et al. (2021)
	The combination of sensory stimulation, multidimensional interaction, and immersive environment enriches my overall emotional experience with the AI-integrated art installation.	Lee and Jung (2014)
	Artworks that skillfully blend artificial intelligence with creative expression deeply resonate with and captivate me.	Akten et al. (2019)

### 3.3 Reliability and construct validity assessment

Both internal consistency testing and exploratory factor analysis (EFA) were conducted to evaluate the psychometric robustness of the survey instrument.

First, reliability was assessed using Cronbach’s alpha. The pilot dataset, analyzed using SPSS version 25, produced an overall Cronbach’s alpha of 0.984, well above the commonly accepted threshold of 0.70 (Nunnally and Bernstein, 1994). As shown in Table 2, all four latent constructs—Sensory Stimuli (SS), Multidirectional Interaction (MI), Immersive Environment (IE), and Emotional Response (ER)—exhibited excellent internal consistency, with alpha coefficients ranging from 0.945 to 0.977. Second, an Exploratory Factor Analysis was conducted on 26 questionnaire items to examine construct validity. The Kaiser–Meyer–Olkin (KMO) measure of sampling adequacy was 0.929, indicating a substantial degree of shared variance among items. Bartlett’s test of sphericity was statistically significant (χ^2^ = 3,633.356, df = 630, *p* < 0.001), confirming the suitability of the dataset for factor analysis. As shown in Table 3, the factor structure revealed a clear and coherent clustering of items, aligning with the proposed dimensional framework of the instrument.

**Table 2:** Reliability statistics (Cronbach’s alpha values).

Variable set	Cronbach’s alpha	Number of items
Multisensory stimuli	0.934	6
Multidirectional interaction	0.959	10
Immersive environment	0.964	11
Emotional responses	0.931	5
total	0.978	32

**Table 3:** Construct validity indicators: KMO and Bartlett’s test.

Kaiser-Meyer-Olkin measure of sample adequacy.		0.929
Bartlett’s test of sphericity	Approx. chi-square	3633.356
	df	630
	Sig.	0.000

Taken together, these findings provide compelling evidence for the questionnaire’s reliability and construct validity. The consistently high Cronbach’s alpha values across all constructs further support the internal coherence of the scale, reinforcing its suitability for application in the main study.

### 3.4 Comparative model evaluation and rationale for selection

In response to reviewer concerns regarding baseline comparisons, a standard linear regression model was incorporated as an additional benchmark. As presented in Table 4, the linear regression model consistently yielded higher mean squared error (MSE) values across all five emotional response dimensions than those observed in nonlinear models, including Random Forests, Support Vector Machines (SVM), and XGBoost. This performance gap highlights the importance of capturing nonlinear feature interactions when predicting affective responses within immersive and interactive environments. Moreover, to enhance transparency and reproducibility, we have clarified in Section 5.6 that five-fold cross-validation (k = 5) was systematically applied to evaluate model generalizability.

**Table 4:** Model comparison of MSE.

LinearRegression	RandomForest	RandomForest(k)	SVM	XGBoost
0.741	0.5643	0.6128	0.53	0.5548
0.5961	0.4797	0.4883	0.5066	0.5031
0.4967	0.4074	0.4332	0.3601	0.4845
0.3691	0.3393	0.3957	0.3403	0.4066
0.4795	0.445	0.4451	0.4292	0.4251

While all nonlinear models outperformed linear regression, Random Forest (RF) demonstrated the most consistent predictive performance across emotional dimensions, particularly excelling in modeling cognitive responses (ER4) and active participation (ER5). SVM showed competitive performance, especially in predicting bodily changes (ER1) and emotional connection (ER3), suggesting a complementary strength between the models. Although XGBoost performed reasonably well, its marginal improvements were offset by longer training times and less interpretability. As such, it was not prioritized for further analysis—details regarding this decision are available in Appendix A.

Given the study’s dual emphasis on model interpretability and alignment with theory-driven design, Random Forest was ultimately selected as the preferred model. Its ability to capture complex, nonlinear interactions while offering clear feature importance rankings makes it well-suited for both predictive accuracy and the derivation of actionable insights in affective art research.

## 4 Data

### 4.1 Participant characteristics and data overview

Microsoft Forms administered an online survey to empirically examine emotional responses to interactive installation art. A total of 623 responses were initially collected. After excluding 157 responses completed in under 3 min and 76 duplicate entries based on predefined quality criteria, the final analytic sample comprised 390 valid responses (63% effective rate). The questionnaire was structured into two main sections: (1) demographic information, including age, gender, education level, and prior exposure to art-related experiences; (2) 36 five-point Likert-scale items designed to measure participants’ emotional and interactive experiences across multiple dimensions.

**Figure 2:**
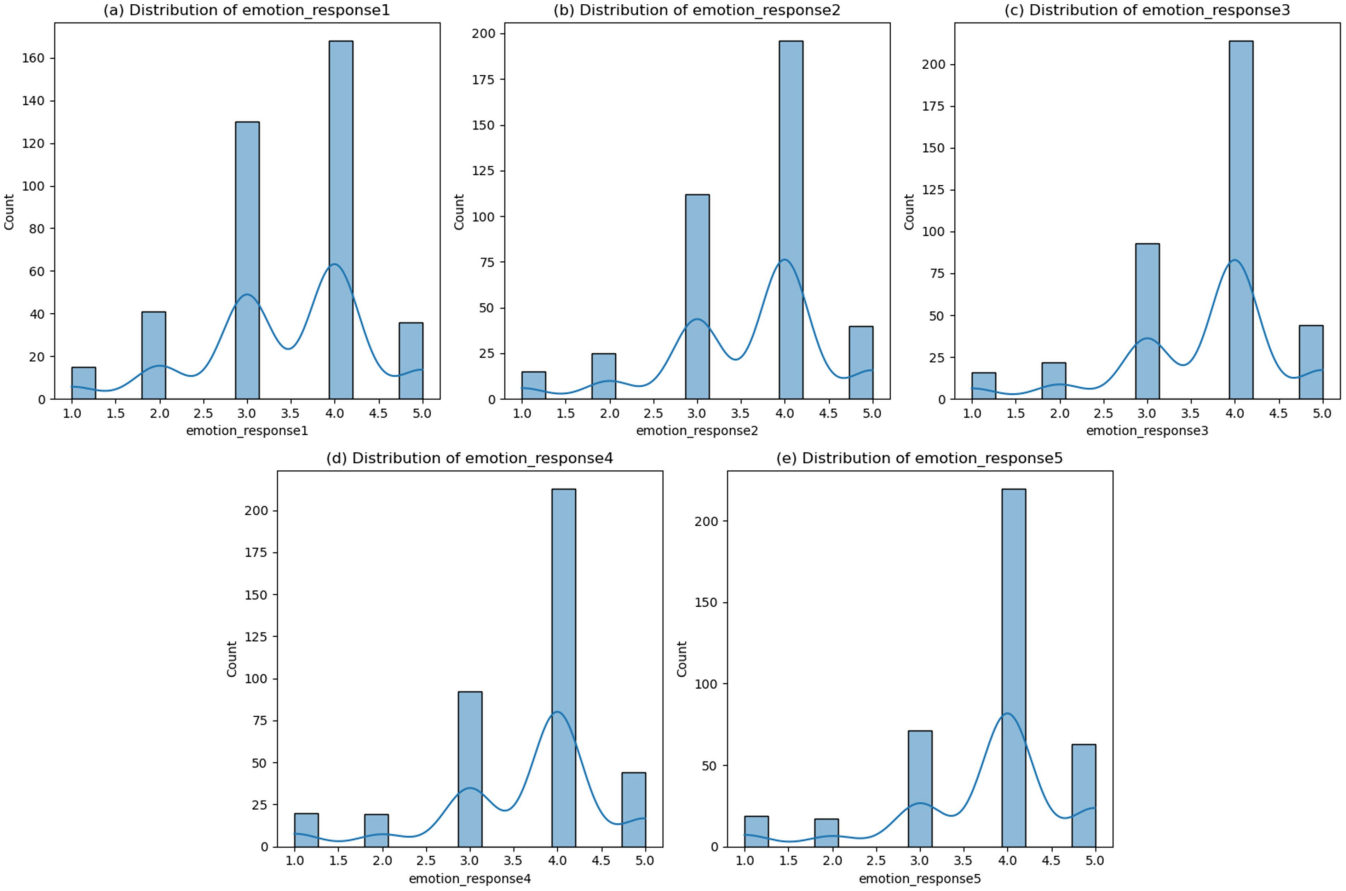
Distribution plots of the five emotional response variables.

As shown in Figure 2, the distribution of emotional response variables was positively skewed, with most responses clustering between 3.5 and 4.0 on the 5-point scale. This suggests that the installations generally elicited moderately strong positive emotional reactions from participants.

A total of 390 participants were included in the final analysis. The sample was composed primarily of young adults, with a mean age of 19.84 years (SD = 2.27). Regarding gender, 284 participants (72.82%) identified as female and 106 (27.18%) as male. Regarding educational background, the vast majority held a Bachelor’s degree (*n* = 381, 97.69%), while a small proportion reported having obtained a Master’s degree (*n* = 2, 0.51%) or a Doctoral degree (*n* = 7, 1.79%). Regarding prior experience in art or a related field, 351 participants (90.00%) indicated relevant exposure, whereas the remaining 39 (10.00%) reported no such experience.

### 4.2 Preprocessing data

Following data collection, a series of preprocessing procedures were implemented to ensure the quality and completeness of the dataset. Prior to analysis, a multi-step data preprocessing procedure was employed. Records with more than 50% missing values were excluded. A small number of missing values were identified in the Likert-scale items and were addressed through mean imputation, whereby the mean of the available responses for each item was calculated and substituted accordingly. This approach included decimal values (e.g., 3.674, 3.78) within the dataset. The total number of imputed values was relatively limited (*n* = 33), and all preprocessing procedures were conducted using SPSS.

For entries with partially missing records, mean imputation was applied based on the empirical distribution of each variable. The independent variables were organized into three conceptual feature sets derived from the theoretical framework:

- SS (Sensory Stimuli): Visual, auditory, and tactile inputs (SS1–SS10)
- MI (Multidimensional Immersion): Spatial control, thematic layering, and narrative structuring (MI1–MI10)
- IE (Immersive Environment): Customization, decision-making, and feedback responsiveness (IE1–IE11)

Table 5 provides descriptive statistics for interaction features and emotional response variables. All three interaction domains exhibited relatively high mean values (ranging from 3.59 to 3.71) and moderate standard deviations (around 0.95–0.99). These findings reflect a generally positive user experience with some inter-individual variability.

**Table 5:** Summary statistics for interaction feature variables and emotional responses.

Variable set	Variable range	Count	Mean	Std Dev
SS variables	SS1 - SS10	388	3.66	0.99
MI variables	MI1 - MI10	387	3.59	0.97
IE variables	IE1 - IE11	386	3.71	0.95
ER	ER1-ER5	389	3.60	0.93

These descriptive patterns suggest that participants perceived the interactive installations as highly immersive, sensory-rich, and conducive to meaningful engagement. These perceptions support the hypothesis that interactive art environments can elicit measurable emotional responses suitable for computational modeling.

## 5 Results

This section presents the results of applying the Random Forest algorithm to predict five distinct dimensions of emotional response elicited by interactive installation art. These dimensions—bodily changes, sensory engagement, emotional connection, cognitive reaction, and active personalization—were operationalized in earlier sections. We report model performance across multiple evaluation criteria, including mean squared error (MSE), feature importance, ROC curves, categorical classification metrics, confusion matrices, and cross-validation analysis.

### 5.1 Mean square error (MSE)

The predictive accuracy of the Random Forest model was first assessed using Mean Squared Error (MSE) for each emotional response dimension. As shown in Table 6, the lowest MSE was observed for Emotional Response 4 (Cognitive Responses, MSE = 0.291), followed by Emotional Response 5 (Active Participation and Personalization, MSE = 0.308). These results suggest that cognitive and participatory reactions exhibit more consistent patterns across individuals, allowing the model to generalize effectively. In contrast, Emotional Response 1 (Bodily Changes) yielded the highest error (MSE = 0.548), highlighting the model’s limitations in capturing the subtle and individualized nature of physiological responses.

**Table 6:** Results of the mean squared error (MSE).

Emotional response	Mean squared error (MSE)
Emotional response 1	0.548
Emotional response 2	0.479
Emotional response 3	0.350
Emotional response 4	0.291
Emotional response 5	0.308

### 5.2 Importance of features

Feature importance was assessed using the Mean Decrease in Impurity (MDI) method, also called Gini importance, which is the default feature ranking technique in the Random Forest implementation of sci-kit-learn (v1.2.2). This method quantifies the total reduction in Gini impurity—aggregated across all trees in the ensemble—attributable to each feature when used as a decision node. A higher Gini importance score reflects greater relevance of the feature in predicting the target variable. To evaluate the unique contribution of predictors across emotional domains, separate Random Forest regression models were trained for each emotional response dimension. Feature importance scores were then systematically extracted from each corresponding model.

**Figure 3:**
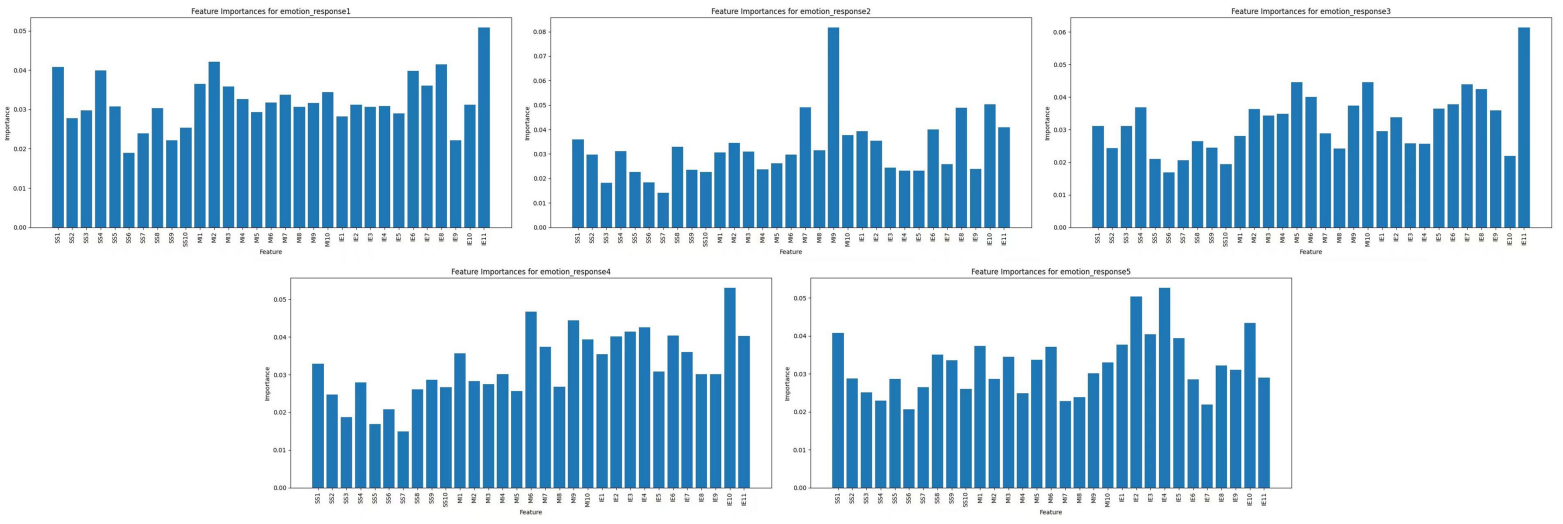
Importance of characteristics for emotional responses.

Feature importance analysis revealed distinct predictors across the five emotional response dimensions (Figure 3). Bodily changes were most influenced by dynamic visual and auditory stimuli—specifically SS2 (Dynamic Color) and MI2 (Sound Feedback)—suggesting that high-intensity sensory inputs are key triggers of physiological reactions such as heart rate fluctuations or posture adjustments. The sensory engagement was primarily shaped by SS5 (Visual–Auditory Coupling) and IE9 (Interaction Freedom), highlighting the role of multimodal coherence and user autonomy in maintaining perceptual attention. Emotional connection was best predicted by MI8 (Emotional Tone of Media), IE3 (Narrative Integration), and SS1 (Subtle Light Variation), emphasizing the importance of affective content and storytelling elements in fostering intimate viewer-artwork relationships. Cognitive responses were driven by SS10 (Conceptual Symbolism), MI1 (Textual Prompting), and IE7 (Decision-Based Interactions), indicating that interpretive depth and user-driven meaning-making are central to analytical engagement. Meanwhile, active participation and personalization were closely linked to IE11 (Customization Options), SS3 (Responsive Surfaces), and MI4 (Real-Time Feedback), underscoring the significance of adaptability and user agency in crafting individualized experiences.

Table 7 further illustrates the recurring influence of several interaction elements—such as SS2, MI8, and IE7/IE11—across multiple emotional domains. This convergence suggests that emotionally rich interaction is not unidimensional but emerges from sensory richness, narrative coherence, and customization flexibility. Designers aiming to optimize emotional engagement in interactive installations may benefit from balancing these core elements to achieve expressive depth and model interpretability.

**Table 7:** Top features by importance for each emotional response variable.

Feature description	PC	SE	EC	CR	AP
Adaptive lighting, dynamic colors, appealing	0.052				
Interface design, personal connection, emotional impact	0.047				
AI techniques, interactive colors, emotional response	0.045				
Real-time responsiveness, diversity, emotional response			0.042		
Layout design, exploration, interaction			0.041		
Complexity, deeper connection, emotional depth		0.047		0.051	
Digital experiences, co-engagement, richer experience			0.042		
Variety of interactions, deepened connection		0.048		0.042	
Adaptive response, amplified impact		0.041	0.049	0.044	
Physical interactions, significant impact		0.04		0.041	0.047
Behavioral adaptation, personalized experience			0.049	0.042	0.042
Visual/auditory feedback, enhanced engagement			0.046		0.065
Game-like elements, enjoyable experience				0.056	0.056
Engagement duration, emotional connection		0.043		0.04	
Responsive interaction, increased engagement	0.047		0.043	0.043	
Sense of loss, profound experience		0.058	0.054		
Emotional intensity, measured engagement		0.067		0.05	
Physical reactions, real-time change, emotional level	0.048		0.056	0.04	

PC = physical changes, SE = sensory engagement, EC = emotional connection, CR = cognitive response, AP = active participation.

### 5.3 The Receiver Operating Characteristic (ROC) curve

The Receiver Operating Characteristic (ROC) curve was used to evaluate the model’s ability to distinguish between categories of emotional responses. The ROC curve illustrates the trade-off between sensitivity and specificity across different decision thresholds by plotting the actual positive rate (TPR) against the false positive rate (FPR). The Area Under the Curve (AUC) serves as a summary metric of classification performance, with higher values reflecting better discriminative capability.

**Figure 4:**
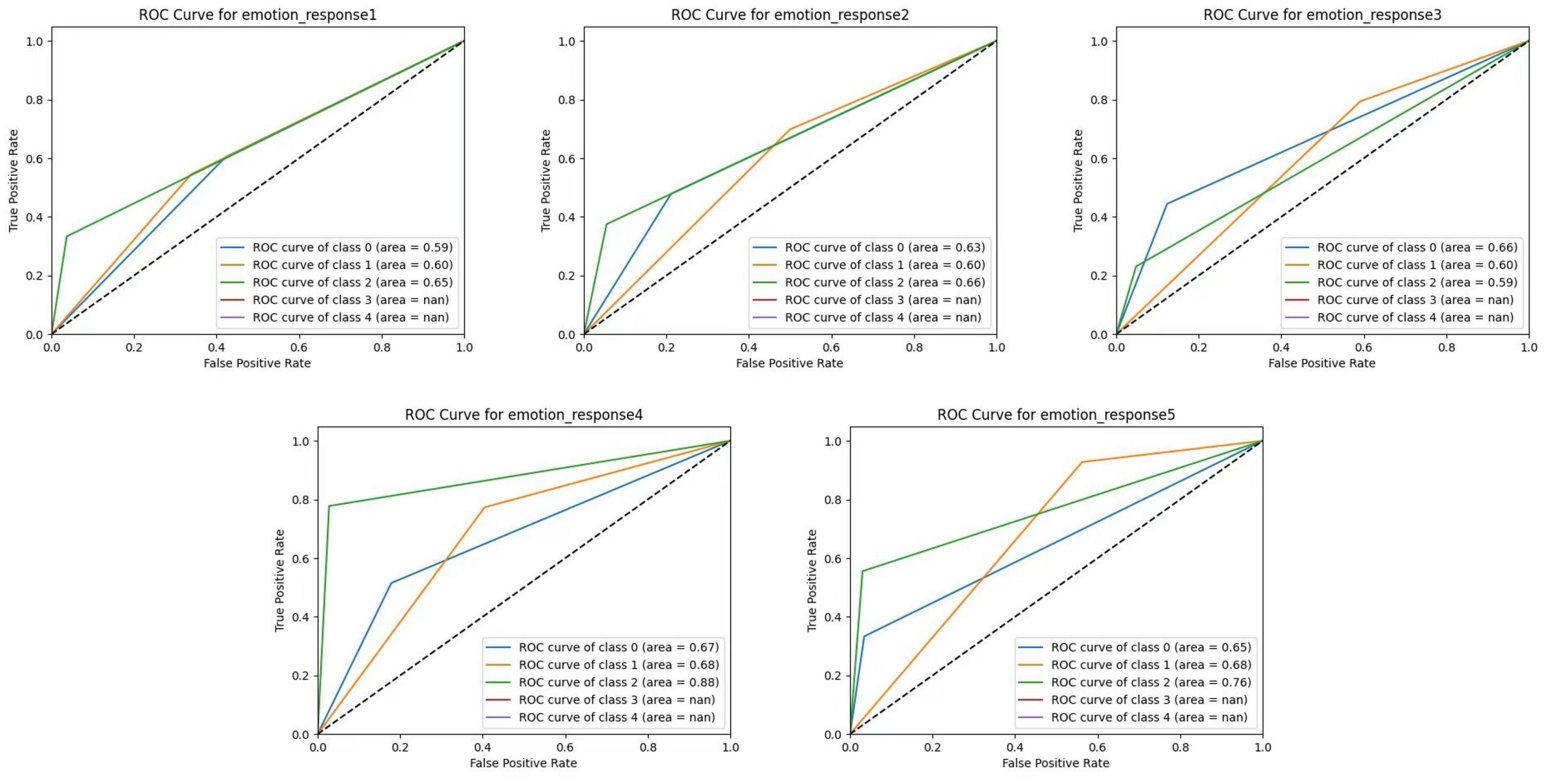
Receiver operating characteristic (ROC) curve.

As shown in Figure 4, the model best predicted Emotional Response 4 (Cognitive Responses), with an AUC of 0.88, suggesting strong sensitivity to interpretation, decision-making, and meaning construction features. Emotional Response 5 (Active Participation and Personalization) also showed favorable results (AUC ≈ 0.83), likely reflecting the model’s ability to capture feature patterns related to customization and user agency. In contrast, lower AUC values were observed for Emotional Response 1 (Bodily Changes) and Emotional Response 3 (Emotional Connection), ranging from 0.59 to 0.66. These reduced scores may stem from the higher individual variability and subjectivity in reporting physical sensations and emotional affinity. Furthermore, the static and self-reported nature of the dataset may limit the model’s ability to capture the dynamic and context-dependent characteristics of these responses.

These results underscore that the model more readily captures structured and cognitively grounded responses, while embodied or affective states require more nuanced input. As highlighted in the feature importance analysis (Section 5.2), decision-based features (e.g., IE7) and semantic elements (e.g., SS10, MI8) appear to be most effective in improving the model’s discriminative power. Future iterations may enhance classification performance—particularly for embodied dimensions—by incorporating temporal, sensor-based, or behavioral interaction data to supplement static questionnaire input.

### 5.4 Categorical evaluation metrics

These variations imply that designing installations to evoke cognitive responses may yield more reliable predictive outcomes. In contrast, bodily and emotional responses may require the integration of sensor-based feedback to improve precision. We evaluated standard classification metrics for each emotional response category to assess model performance, including accuracy, precision, recall, and F1-score. Precision quantifies the proportion of true positives among all predicted positives, while recall reflects the proportion of correctly identified positive cases. The F1-score represents the harmonic mean of precision and recall, providing a balanced measure of predictive performance.

As shown in Table 8, the model performs best in predicting Emotional Response 4 (Cognitive Responses), achieving a precision of 0.70, recall of 0.692, and an F1-score of 0.675. These results are consistent with the ROC analysis and suggest that the model can effectively capture reflective and analytical engagement patterns. In contrast, Emotional Response 1 (Bodily Changes) exhibits the lowest performance across all metrics, with an F1-score of only 0.411. This indicates a challenge in accurately detecting subtle physical responses, which may be less consistently reported or inadequately captured by the available features. Emotional Responses 2, 3, and 5 show intermediate performance, with F1-scores ranging from 0.524 to 0.629, suggesting varying levels of model reliability across affective dimensions.

**Table 8:** Categorical evaluation indicators.

Emotional response	Accuracy	Precision	Recall	F1-score
Emotional response 1	0.436	0.404	0.436	0.411
Emotional response 2	0.564	0.502	0.564	0.524
Emotional response 3	0.603	0.617	0.603	0.554
Emotional response 4	0.692	0.7	0.692	0.675
Emotional response 5	0.667	0.645	0.667	0.629

These variations highlight the differential predictability of emotional responses, suggesting that cognitive states may be more stable and feature-dependent. In contrast, bodily and affective reactions require richer contextual or physiological input for accurate modeling. Cognitive responses appear more stable and feature-dependent, whereas bodily and affective responses may require more sensor-based or dynamic input data to enhance prediction accuracy. From an application standpoint, these findings suggest that interactive installations that elicit cognitive engagement—such as contemplation, meaning-making, or problem-solving—can be effectively optimized using machine learning-based prediction. However, installations targeting embodied or emotional resonance may benefit from integrating real-time physiological or behavioral data to improve responsiveness and personalization.

### 5.5 Confusion matrix

**Figure 5:**
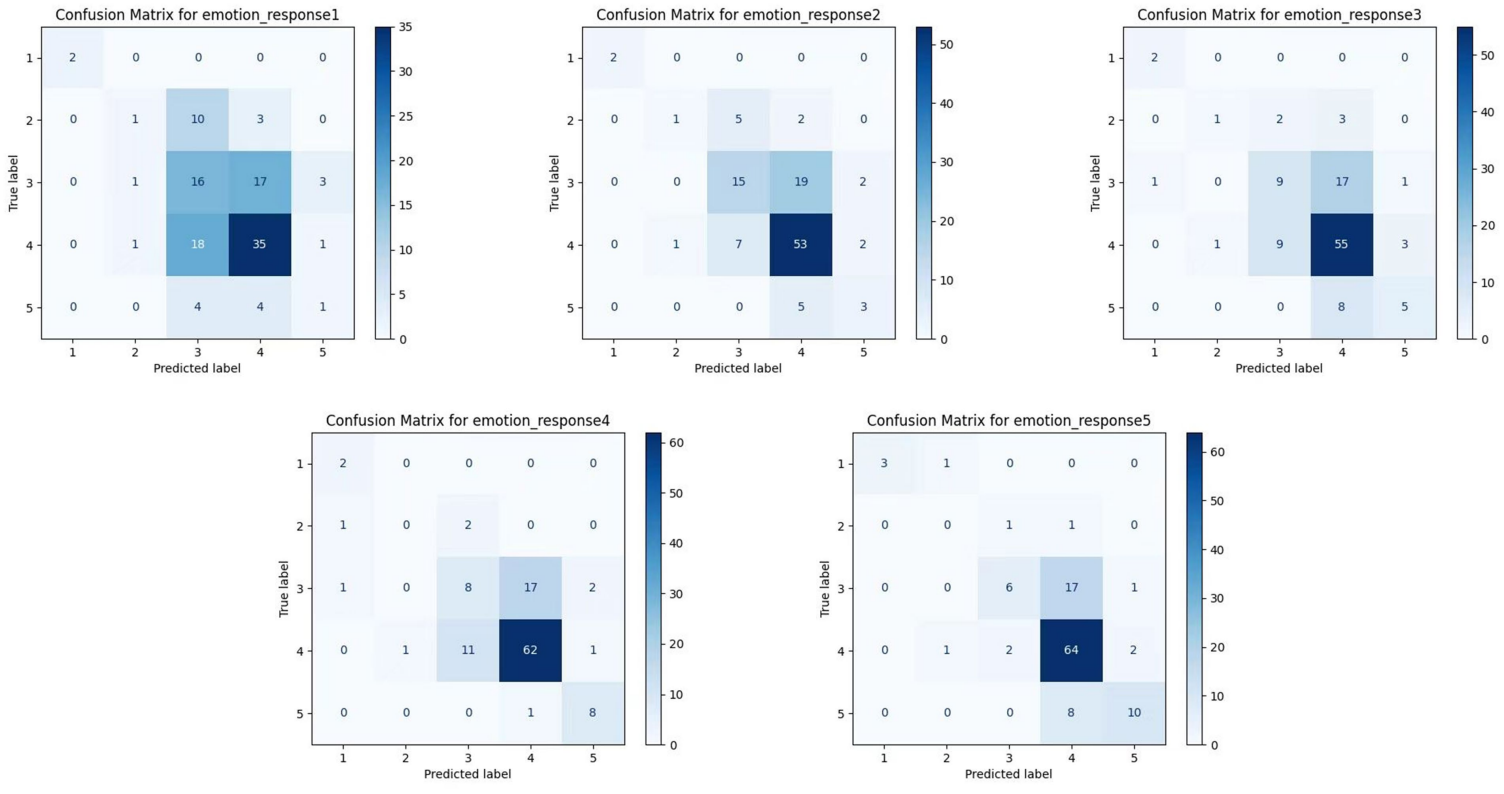
Confusion matrix.

We examined confusion matrices for each emotional response category to further assess model performance at the class level, as shown in Figure 5. Each matrix presents the distribution of predicted labels versus actual labels, highlighting patterns of correct classification and systematic misclassification.

The model demonstrates the strongest classification performance for Emotional Response 4 (Cognitive Responses), with 62 out of 82 instances correctly predicted as Class 4. This aligns with previous ROC and F1-score analysis findings, confirming the model’s ability to capture cognitive engagement features accurately. Similarly, Emotional Response 5 (Active Participation and Personalization) shows robust performance, with 64 true positives, suggesting that user-driven customization is effectively modeled. In contrast, classification performance for Emotional Response 1 (Bodily Changes) is notably weaker. The model frequently confuses this class with Class 3 and Class 4, indicating difficulty distinguishing bodily reactions from emotional or cognitive responses. A similar confusion is observed for Emotional Response 3 (Emotional Connection), where predictions often spill over into adjacent categories, especially Class 4.

These misclassifications suggest overlapping or insufficiently distinct feature representations among certain emotional states—particularly those involving subtle, internalized experiences such as physical sensation and emotional bonding. From a modeling standpoint, these results indicate that while the current feature set adequately distinguishes cognitive and active involvement responses, it may lack sensitivity for more embodied or affective dimensions. Future iterations could benefit from integrating real-time biometric data (e.g., heart rate, galvanic skin response) or temporal interaction metrics to isolate these nuanced states better. From a design perspective, the observed confusion between emotional and bodily responses highlights the need for more differentiated stimuli and feedback strategies. For instance, installations that evoke physical responses might emphasize kinesthetic interaction or sensory overload, whereas those aiming for emotional connection could benefit from narrative coherence or affective content. Such insights guide model enhancement and inform the design of emotionally differentiated interactive installations.

### 5.6 Cross-validation

To assess the generalizability and robustness of the Random Forest model, 5-fold cross-validation (k = 5) was employed. This technique is widely recognized for its effectiveness in mitigating overfitting and balancing the trade-off between bias and variance when estimating model performance on mid-sized datasets. The cross-validation procedure thus enables a comprehensive evaluation of model performance across the training set, validation folds, and an independent test set. Specifically, the dataset was partitioned into five equally sized subsets. Each subset was used once as a validation fold, while the remaining four subsets were used to train the model. This process was repeated five times, and the results were averaged across all folds to produce a more robust and generalizable performance estimate. Table 9 presents the evaluation metrics—accuracy, precision, recall, and F1 score—computed for each emotional response category at each evaluation stage, including training, cross-validation, and testing.

**Table 9:** Comparison of model evaluation metrics for emotional responses.

	Training set				Cross-validation	Test set			
Emotional response	Accuracy	Precision	Recall	F1-Score	Accuracy ± Std	Accuracy	Precision	Recall	F1-Score
Emotional response 1	0.436	0.404	0.436	0.411	0.490 ± 0.036	0.397	0.421	0.397	0.379
Emotional response 2	0.564	0.502	0.564	0.524	0.638 ± 0.018	0.551	0.529	0.551	0.523
Emotional response 3	0.603	0.617	0.603	0.554	0.634 ± 0.045	0.59	0.571	0.59	0.543
Emotional response 4	0.692	0.7	0.692	0.675	0.667 ± 0.034	0.769	0.74	0.769	0.746
Emotional response 5	0.667	0.645	0.667	0.629	0.680 ± 0.035	0.705	0.68	0.705	0.673

Consistent with earlier findings, Emotional Response 4 (Cognitive Responses) achieves the highest accuracy and stability across all stages, with a cross-validation accuracy of 0.667 (±0.034) and a test set accuracy of 0.769. Similarly, Emotional Response 5 (Active Participation and Personalization) maintains strong and stable performance across data splits. These results underscore the model’s capacity to reliably capture patterns related to reflective engagement and user-driven interaction, which are likely more consistent and well-defined in the data. In contrast, Emotional Response 1 (Bodily Changes) shows the lowest accuracy and F1 score across all sets, with a cross-validation accuracy of 0.490 (±0.036) and a test accuracy of 0.397. This again reflects the challenge of modeling physical responses, which may be subject to more significant individual variation and ambiguity in self-reporting.

Overall, the relatively low standard deviations across cross-validation folds suggest that the model exhibits stable performance and is not overly dependent on particular subsets of the training data. However, the discrepancy between emotional categories highlights the importance of feature representation quality. Bodily and emotional responses may highlight the potential benefit of incorporating richer temporal or sensor-based features in future modeling efforts.

## 6 Discussion

### 6.1 Summary of findings

This study employed a Random Forest (RF) algorithm to model and predict five distinct dimensions of emotional responses evoked by interactive installation art. Among these dimensions, the model demonstrated the highest predictive performance for Emotional Response 4 (Cognitive Responses) and Emotional Response 5 (Active Participation and Personalization), yielding test accuracies of 0.769 and 0.705 and corresponding F1-scores of 0.746 and 0.673, respectively. These results indicate that responses involving reflection, interpretation, and user agency tend to follow more consistent and detectable patterns across individuals, making them well-suited for computational prediction. In contrast, the model exhibited considerably lower predictive performance for Emotional Response 1(Bodily Changes), with an F1-score of 0.379 and an accuracy of 0.397. This finding suggests that embodied and physiological reactions are more idiosyncratic and difficult to capture through symbolic or self-reported indicators alone, such as Likert-scale questionnaire ratings, keyword tagging, or post-experience reflection logs, which may lack the temporal granularity and sensorimotor precision needed to represent real-time bodily responses. The moderate performance observed for Emotional Response 2 (Sensory Engagement) and Emotional Response 3 (Emotional Connection) further highlights the modeling challenges posed by affective states characterized by subjectivity, pre-reflective qualities, or subtle experiential cues.

Model performance remained stable across training, cross-validation, and test sets, demonstrating satisfactory generalizability and robustness. These findings collectively suggest that emotion types grounded in cognitive appraisal, intentional engagement, and user-system interaction are more amenable to structured prediction. At the same time, those rooted in sensorimotor or affective processes may require richer, multimodal, or temporal data to enhance predictive precision.

### 6.2 Comparison with existing literature

This study’s differentiated predictive performance across emotional response dimensions is broadly consistent with existing theoretical and empirical research on affective engagement in interactive art. Pelowski et al. (2018) proposed a stratified model of art experience encompassing sensory, emotional, and cognitive tiers—a conceptual structure echoed in the layered nature of the current model’s performance. Specifically, the high predictability of Emotional Response 4 (Cognitive Responses) reinforces previous findings that reflective engagement and symbolic interpretation yield more structured and measurable emotional outcomes.

The significant predictive contribution of features such as customization (IE11) and conceptual richness (MI8) further supports the findings of Savaş et al. (2021), who underscored the importance of participatory agency and immersive content in intensifying emotional involvement. Similarly, Capece and Chivăran (2020) emphasized the emotional salience of sensorial complexity and adaptive feedback, a notion mirrored in our results through the prominent roles of SS2 (dynamic lighting) and SS5 (multisensory coupling). These results align with the observations of Meinshausen (2006), who demonstrated that environmental parameters—such as spatial geometry and illumination—play a central role in modulating affective arousal during immersive experiences. This correspondence also resonates with the sensory dimensions posited by Russell’s Circumplex Model of Affect. Conversely, the model’s comparatively weak performance in predicting Emotional Response 1(Bodily Changes) reflects a recurring challenge in the literature. Baranauskas and Duarte (2024) noted that embodied affective states often elude precise prediction due to their inherently pre-reflective and idiosyncratic nature. Meinshausen (2006) and others have suggested that such phenomena may require the integration of physiological indicators (e.g., EEG, HRV) to overcome the limitations of symbolic and self-report data—a methodological limitation acknowledged in our current study and one that warrants future multimodal extensions.

From a theoretical perspective, Kragel and LaBar (2016) have posited that emotional states are best conceptualized as discrete, high-dimensional neural representations rather than simplified valence-arousal coordinates. This framework provides a plausible rationale for the strong predictability of features linked to symbolic meaning-making, user agency, and cognitive control—constructs manifest in more stable and computationally tractable patterns. In contrast, affective states grounded in somatosensory feedback and proprioceptive input are inherently more variable, thus complicating their algorithmic modeling. Moreover, our findings lend empirical support to enactive aesthetics (Gallagher, 2005; Varela et al., 1991) and affective loop theory (Höök, 2008), emphasizing the co-constructive dynamics between system and user in shaping emotional meaning. The model’s robust performance in predicting reflective responses suggests that emotionally meaningful interaction emerges most reliably when engagement is intentional, reciprocal, and semantically rich. These observations further advocate for a paradigmatic shift—from viewing interactive art as a static object of reception to understanding it as a dynamic, relational system capable of eliciting coherent affective trajectories (Pelowski et al., 2018).

Given these findings—particularly the model’s strong performance in predicting cognitively and symbolically grounded responses—it becomes evident that symbolic and interpretable features offer a viable alternative to data-intensive deep learning methods when theoretically mapped and systematically selected. While approaches such as those by Hossain and Muhammad (2019) excel with audiovisual big data, our results reaffirm that theory-driven, semantically rich models can achieve comparable predictive efficacy, especially in contexts where explainability and psychological grounding are critical, such as emotionally responsive and experience-centered design.

### 6.3 Complementarity of models and justification for random forest adoption

The comparative analysis between the Random Forest (RF) and Support Vector Machine (SVM) models revealed a pattern of complementary strengths across the five emotional response dimensions. Although SVM achieved a marginally lower mean squared error (MSE) in predicting emotional connection (0.3601 vs. 0.4074 for RF), the RF model exhibited more stable and balanced performance across all dimensions, particularly in cognitive reflection and active personalization. However, as no statistical significance tests were conducted, these differences should be interpreted as suggestive rather than conclusive.

One of the primary motivations for selecting the RF model lies in its advantageous balance between predictive reliability and interpretability—an essential consideration for theory-driven, exploratory research. The RF algorithm provides feature importance rankings, which allow researchers to trace how specific interaction design variables—such as dynamic sensory stimuli, narrative structure, and customization affordances—contribute to variations in emotional responses. Such transparency is critical when the research goal includes prediction and explanatory insight, particularly in the context of complex, multisensory experiences like interactive art. This interpretive capacity is particularly valuable for generating initial, non-definitive insights into the mechanisms by which interactive installations evoke emotional engagement.

The divergent performance trends observed between RF and SVM further suggest that each model may capture distinct aspects of emotional variability. Whereas RF appears more sensitive to generalizable, design-level patterns, SVM may be better equipped to detect boundary cases and finer-grained deviations. This complementarity implies that model selection should be guided by overall performance metrics and alignment with specific research goals—whether interpretability, generalizability, or sensitivity to extremes. While these inferences are promising, they remain speculative without additional empirical validation, and no claims are made regarding the overall superiority of either model.

Both models are well-suited for structured, tabular datasets; however, their relative strengths and weaknesses become more evident when extended to complex multimodal inputs, such as interaction logs or sensor-derived physiological features. RF’s robustness to noise and its moderate scalability render it a practical choice in such contexts, though its performance may still benefit from further feature engineering. In contrast, SVM is potentially more vulnerable to performance degradation under these conditions. Thus, the comparative evaluation highlights the potential utility of ensemble or hybrid approaches that strategically integrate the respective strengths of both models.

In sum, the adoption of RF in the present study reflects a broader methodological orientation within affective computing that seeks to integrate interpretability, theoretical coherence, and predictive competence. Given the inherently dynamic, co-constructed, and sometimes ambiguous nature of user responses in interactive installation art, transparent models such as RF may offer pragmatic value as exploratory tools. While not definitive, these initial findings support the application of RF in similar research contexts, and they highlight the need for future investigations involving larger samples and rigorous statistical validation to enhance generalizability.

### 6.4 Design implications and application

The findings of this study yield preliminary, theory-driven insights that may inform the design of emotionally responsive interactive installations. While the Random Forest model identified salient predictors across five emotional response dimensions, these results should be interpreted as exploratory rather than prescriptive. The observed associations offer potential guidance for design strategies to elicit targeted emotional outcomes, although additional empirical work is needed to confirm underlying causal mechanisms.

To foster bodily engagement (e.g., physiological activation), designers may consider incorporating synchronized multisensory components—such as dynamic lighting, haptic feedback, and immersive audio environments—that have been theorized to enhance corporeal awareness and embodied affective states. In contrast, cognitive responses such as reflection and interpretation may be better supported by integrating abstract narrative prompts, symbolic metaphors, and decision-driven pathways. These design strategies are conceptually grounded in research on embodied cognition and participatory aesthetics, emphasizing the user’s active role in constructing meaning through sensory and symbolic interaction.

Concerning emotional connection, two complementary design strategies can be delineated. First, emotionally coherent storytelling and expressive dramaturgical structures may strengthen empathic resonance. Second, adaptive feedback mechanisms—such as real-time visual cues or context-aware system responses—can adjust interaction dynamics based on the user’s emotional state. These techniques align with affective loop models, which conceptualize emotion as a dynamic feedback process between user and system; however, their empirical efficacy in interactive art contexts requires further validation.

Additional design features such as user-customizable interfaces and real-time interactivity may enhance perceived agency and emotional investment, which are foundational to participatory and user-centered interaction. Nonetheless, the extent to which such features exert a causal influence on emotional outcomes remains an open empirical question, warranting further exploration in controlled experimental settings.

Beyond theoretical exploration, the results suggest several potential—though not yet definitive—applications:

- Museum and Exhibition Design: Emotion-informed interaction strategies could inform curatorial planning, enabling the shaping of emotionally resonant visitor experiences.
- Therapeutic and Clinical Contexts: Emotion-sensitive installations may offer exploratory value in mental health interventions or emotion regulation training, although clinical efficacy must be systematically assessed.
- Commercial and Experiential Environments: Interactive systems with basic affective responsiveness could be leveraged to support personalized engagement, particularly in brand storytelling and consumer experience design domains.

These possible implementations are within broader theoretical frameworks in affective computing and neuroscience-informed design, which conceptualize emotion as a multidimensional, embodied, and contextually modulated phenomenon (Kragel and LaBar, 2016). The comparatively stronger predictive performance observed in cognitively mediated responses—such as reflection and personalization—may reflect their greater structural consistency and interpretability, suggesting that specific emotional dimensions are more amenable to computational modeling.

In conclusion, while the present study does not offer prescriptive design solutions, it contributes to a growing empirical foundation for emotion-oriented interaction design. Future research should adopt interdisciplinary approaches—combining computational modeling, iterative design experimentation, and longitudinal validation—to realize the potential of emotionally adaptive systems across cultural, clinical, and commercial settings.

### 6.5 Theoretical contributions

This study advances the theoretical discourse on affective interaction by contributing to two interrelated domains: enactive aesthetics and participatory emotion modeling. The model’s strong predictive performance for cognitive and participatory emotional responses offers empirical reinforcement for frameworks that emphasize user agency, meaning-making, and the co-construction of emotional experience (Domingues et al., 2014; Teo et al., 2019). Crucially, successfully implementing a feature-driven predictive framework establishes a conceptual bridge between computational modeling and core psychological constructs such as intentionality, interpretive engagement, and reflective consciousness. These findings underscore the potential of machine learning to empirically ground abstract theoretical ideas, enabling a richer dialogue across disciplines such as human-computer interaction (HCI), affective computing, and digital aesthetics.

Furthermore, the observed predictive gradient—in which symbolic and agency-related responses (e.g., cognitive and participatory engagement) were modeled with higher accuracy than embodied or affective responses—demonstrates the utility of a mapping-based theoretical approach. The study highlights that some emotional states follow more structured, repeatable, and computationally learnable patterns by aligning interactional features with established emotion theories, such as Russell’s Circumplex Model and the Affective Loop framework. In addition, this work contributes to the expanding literature on computational aesthetics, showcasing how interpretable and theoretically anchored model outputs can support both scientific analysis and design-driven decision-making. The predictive framework introduced here facilitates emotion modeling in immersive environments and informs the development of emotionally intelligent systems grounded in well-articulated psychological theory.

Looking ahead, this symbolic and semantically rich modeling approach holds promise for visualizing affective dynamics, such as generating heatmaps of emotional resonance across interactive spaces or simulating user emotion trajectories over time. Such visual analytics could offer designers, curators, and researchers new tools for interpreting the emotional topography of interactive installations—further reinforcing the theoretical-practical synergy underpinning this research.

### 6.6 Limitations and future research

Despite offering valuable insights into the predictive modeling of emotional responses to interactive art, this study is subject to certain limitations.

First, the data relied exclusively on self-reported emotional responses. While self-report instruments are standard in affective computing research, they are inherently limited in capturing embodied, pre-reflective experiences—particularly those associated with bodily changes. The model’s relatively low performance in predicting Emotional Response 1 (Bodily Changes) likely reflects the subjective nature and limited granularity of such self-reports, which are often shaped by individual introspective ability and contextual interpretation. Second, although the Random Forest algorithm demonstrated robust and interpretable performance, it is less effective in modeling temporal dynamics or latent emotional processes that evolve. In contrast, deep learning approaches—such as convolutional neural networks (CNNs) and long short-term memory networks (LSTMs)—have shown greater efficacy in extracting spatiotemporal features from sequential and multimodal data. However, this advantage often comes at the cost of model transparency and interpretability, which are essential in theory-driven design contexts. Third, the generalizability of our findings is constrained by the specific research context: a selected group of interactive installation artworks and a relatively homogeneous participant sample. Emotional responses to interactive art may vary substantially across different media types, cultural backgrounds, and interaction modalities, limiting the current model’s external validity and broader applicability.

Fourth, the sample primarily consisted of university students (mean age = 19.84), with a gender distribution slightly skewed toward female participants. Although male participants were represented in non-negligible numbers, this demographic imbalance may have introduced bias and constrained the generalizability of the findings to more diverse populations. To enhance external validity, future research should strive to include a broader age range and a more balanced gender composition. Finally, while the built-in feature importance scores provided by Random Forest offer valuable insights into the relative contribution of input variables, they do not convey information regarding statistical significance. This limitation reduces the interpretability of model outputs and constitutes a methodological constraint of the present analysis. Future studies could address this issue by incorporating permutation-based importance measures or SHAP (Shapley Additive exPlanations) values to more rigorously assess the robustness and statistical reliability of the identified predictors. To address these limitations, future research may consider the following directions:

- Multimodal data integration: Incorporating physiological signals such as heart rate, galvanic skin response, or eye-tracking data could enrich our understanding of affective states and capture preverbal or embodied emotional dimensions more effectively.
- Temporal modeling: Leveraging time-series analysis or recurrent neural networks could allow for the detection of dynamic emotional transitions, particularly within critical “emotion windows” or micro-interaction moments that shape the unfolding of affective experiences. Capturing these short-term fluctuations can enhance the temporal granularity of prediction and support more context-aware, real-time adaptation in interactive environments.
- Model comparison and hybrid approaches: Comparative analyses between interpretable models (e.g., Random Forests and SVMs) and deep learning methods—or their integration in ensemble architectures—could balance predictive accuracy with theoretical insight.
- Audience segmentation: Adapting models to user-specific attributes such as prior artistic experience, interaction style, or personality traits may enhance model sensitivity and inclusiveness. Additionally, incorporating sociocultural variables—such as age, gender identity, or cultural background—can account for contextual diversity in emotional perception and help ensure that emotion-aware systems remain equitable and representative across heterogeneous user populations.

As discussed in the final section, these directions address the current study’s limitations and lay a methodological foundation for broader research trajectories. By engaging with these avenues, future studies can advance both the theoretical and practical frontiers of emotion modeling in interactive systems—enabling the development of more adaptive, context-aware, and ethically responsible effective technologies.

## 7 Conclusion

Using a Random Forest model, this study proposed a data-driven framework for predicting emotional responses to interactive installation art. Grounded in theories of human-computer interaction and affective computing, the modeling approach was designed to align interactional variables with core emotional mechanisms. Drawing on 390 valid responses from a questionnaire-based survey, we examined how sensory stimulation, immersive media, and interactive engagement predict five distinct emotional response dimensions: bodily changes, sensory engagement, emotional connection, cognitive reflection, and active personalization.

The model demonstrated the strongest predictive accuracy for cognitive responses and active participation, with F1 scores exceeding 0.67 and a low MSE of 0.291 for cognitive responses. In contrast, predictions for bodily changes were considerably less accurate, underscoring the complexity and subjective variability of embodied emotional states. Such states often involve subtle and pre-reflective physiological phenomena—such as muscle tension, respiration, or thermoregulation fluctuations—that are difficult to verbalize or quantify using symbolic or self-reported measures alone (Damasio, 1994; Niedenthal, 2007). This distinction aligns with the framework of embodied cognition, which posits that emotional meaning is rooted in bodily experiences and sensorimotor feedback loops that precede conscious appraisal (Niedenthal, 2007). Moreover, it supports the view that symbolic, appraisal-based emotional constructs—such as intentional reflection and user agency—tend to yield more consistent and computationally learnable patterns (Kragel and LaBar, 2016).

By integrating insights from affective computing, empirical aesthetics, and interactive system design, this work demonstrates the feasibility of using interpretable machine learning models to inform emotion-aware design strategies. These models are theory-aligned—informed by established frameworks such as Russell’s Circumplex Model of Affect, Affective Loop Theory, and Enactive Aesthetics—which collectively emphasize the dynamic, co-constructed nature of emotional meaning. The ability to link specific interaction features (e.g., personalization, narrative depth, multisensory coupling) to emotional outcomes thus provides both empirical grounding and actionable implications for creating emotionally adaptive, user-centered art environments.

Future research should aim to advance the development of emotionally intelligent systems that can detect and adaptively respond to user affect in context-sensitive and ethically attuned ways. Such systems should move beyond isolated affect detection to support dynamic emotional co-regulation and longitudinal effective modeling—enabling real-time content adaptation based on evolving user profiles and emotional trajectories. Future studies may explore applications across diverse experiential domains, including immersive education, public cultural engagement, and therapeutic environments, to realize this vision. These contexts present unique emotional dynamics and social expectations, offering fertile ground for validating emotion-aware technologies in real-world settings. Additionally, emerging challenges such as emotional privacy, algorithmic bias, and the need for transparent, effective inference mechanisms warrant closer attention. Integrating concepts from affective ethics, emotional memory modeling, and cross-modal interpretability will ensure that emotionally responsive systems remain inclusive, respectful, and human-centered.

Ultimately, the proposed framework offers a promising step toward developing emotionally intelligent interactive installations—systems capable of dynamically adapting content based on users’ inferred emotional states. Such advancements hold potential for applications in art therapy, immersive learning, and public engagement, creating more inclusive, emotionally resonant interactive experiences and paving the way toward a new generation of emotionally attuned, human-centered design practices.

## Data Availability

The original contributions presented in the study are included in the article/Supplementary material, further inquiries can be directed to the corresponding author/s.

## Appendix A

### Rationale for excluding XGBoost from final model comparison

While XGBoost is widely recognized for its predictive power in affective computing and related domains, it was excluded from the final comparative analysis due to its disproportionately high computational cost and marginal performance gains. Early trials using the same dataset and feature set revealed that XGBoost required significantly longer training times (up to 3.5 h per target dimension) compared to under 1 h for Random Forest or SVM, without meaningful improvements in MSE. In several cases, the model failed to converge under the available computational constraints (Intel i7-10750H, 16GB RAM). This observation is consistent with prior research (Chen and Guestrin, 2016; Llewelyn et al., 2023), which highlights the sensitivity of boosting models to dataset scale and feature redundancy. Given the study’s dual emphasis on predictive validity and interpretability, Random Forest emerged as the more suitable model due to its faster training and transparent feature importance ranking. SVM was retained for benchmarking due to its distinct performance profile and computational efficiency. Future work may revisit XGBoost or incorporate deep learning models should larger datasets and enhanced hardware environments become available.
